# Revisiting racial differences in ESRD due to ADPKD in the United States

**DOI:** 10.1186/s12882-019-1241-1

**Published:** 2019-02-14

**Authors:** Erin L. Murphy, Feng Dai, Katrina Lehmann Blount, Madeline L. Droher, Lauren Liberti, Deidra C. Crews, Neera K. Dahl

**Affiliations:** 1 Section of Nephrology, Yale University School of Medicine, New Haven, CT 06520 USA; 2 Biostatistics, Yale University School of Public Health, New Haven, CT 06511 USA; 3 Division of Nephrology, Department of Medicine, Johns Hopkins University School of Medicine, Baltimore, MD USA

**Keywords:** Autosomal dominant polycystic kidney disease (ADPKD), End-stage renal disease (ESRD), Incidence, United States renal data system (USRDS)

## Abstract

**Introduction:**

Autosomal dominant polycystic kidney disease (ADPKD) affects all races. Whether the progression of ADPKD varies by race remains unclear.

**Methods:**

In this retrospective cohort study from 2004 to 2013 non-Hispanic blacks and non-Hispanic whites of all ages classified in the US Renal Data System (USRDS) with incident ESRD from ADPKD (*n* = 23,647), hypertension/large vessel disease (*n* = 296,352), or diabetes mellitus (*n* = 451,760) were stratified into five-year age categories ranging from < 40 to > 75 (e.g., < 40, 40–44, 45–49, …, 75+). The Cochran-Mantel-Haenszel test was used to determine the association of race and incidence of ESRD from ADPKD, diabetes, or hypertension. The difference in the proportions of ESRD in non-Hispanic black and non-Hispanic white patients at each age categorical bin was compared by two-sample proportion test. The age of ESRD onset between non-Hispanic black and non-Hispanic white patients at each year was compared using two-sample t-test with unequal variance.

**Results:**

1.068% of non-Hispanic blacks and 2.778% of non-Hispanic whites had ESRD attributed to ADPKD. Non-Hispanic blacks were less likely than non-Hispanic whites to have ESRD attributed to ADPKD (odds ratio (OR) (95% CI) = 0.38 (0.36–0.39), *p* <  0.0001). Using US Census data as the denominator to adjust for population differences non-Hispanic blacks were still slightly under-represented (OR (95% CI) 0.94 (0.91–0.96), *p* = 0.004). However, non-Hispanic blacks with ADPKD had a younger age of ESRD (54.4 years ±13) than non-Hispanic whites (55.9 years ±12.8) (*p* <  0.0001). For those < 40 years old, more non-Hispanic blacks had incident ESRD from ADPKD than non-Hispanic whites (9.49% vs. 7.68%, difference (95% CI) = 1.81% (0.87–2.84%), *p* <  0.001) for the combined years examined.

**Conclusions:**

As previously shown, we find the incidence of ESRD from ADPKD in non-Hispanic blacks is lower than in non-Hispanic whites. Among the younger ADPKD population (age < 40), however, more non-Hispanic blacks initiated dialysis than non-Hispanic whites. Non-Hispanic blacks with ADPKD initiated dialysis younger than non-Hispanic whites. A potential implication of these findings may be that black race should be considered an additional risk factor for progression in ADPKD.

## Introduction

The prevalence of autosomal dominant polycystic kidney disease (ADPKD) ranges from 1:400 to 1:1000 [1]. This heterogeneous disorder is a leading cause of end-stage renal disease (ESRD) affecting 12.5 million people globally and is the 4th leading cause of ESRD [2, 3].

Within the US, there are few ADPKD studies or registries that contain greater than 200 participants and clearly document subject race. Both the Consortium for Radiologic Imaging Studies of Polycystic Kidney Disease (CRISP) cohort [4] and the HALT Polycystic Kidney Disease (HALT-PKD) trials [5] are large studies which provide this information. On average, according to the 2000 and 2010 US Census, approximately 76% of the US population identify as white (percent reporting only 1 race), while approximately 13% identify as black (percent reporting only 1 race). In CRISP, whites and blacks compose 86.7 and 11.6% (27/241) of the cohort, respectively, reflecting the US population [4]. In each arm of HALT-PKD (study A, standard – patients with higher eGFRs and standard blood pressure targets, study A, low – patients with higher eGFRs and low blood pressure targets, and study B – patients with lower eGFRs), there are fewer black ADPKD patients than would be expected relative to general US population (2.5, 2.6, and 2.5%, respectively) [5]. Thus, both HALT and CRISP may underestimate a potential impact of race on progression of ADPKD because of low numbers of blacks in the studies.

The PROPKD score uses urologic event and/or hypertension before the age of 35, genetic mutation (truncating *PKD1*, non-truncating *PKD1* or *PKD2*) and gender to determine the risk of progression to ESRD [6]. The patients enrolled in the reference database were primarily Caucasian [7]. An alternative risk calculator, based on height-adjusted total kidney volume (htTKV), age, gender and creatinine, was developed based on data from the CRISP cohort and Mayo database [8]. In this model, race was used to calculate eGFR, but race did not affect risk of progression [9]. However, there were relatively few black patients in the analysis which may mask a potential difference in disease progression impacted by race. Because these risk calculators will be increasingly used to determine risk of progression in ADPKD and subsequently, treatment eligibility, it is important to examine whether race may be an additional possible risk factor for disease progression.

There is data suggesting that race may impact risk of progression to ESRD in ADPKD. Yium et al. found that sickle cell hemoglobin trait occurred more frequently in blacks having ESRD secondary to ADPKD, and these patients had earlier onset of ESRD than black ADPKD patients with normal hemoglobin. After adjusting for population differences, the incidence of ADPKD in blacks and whites were similar (0.48 versus 0.47 per 100,000 population), with an earlier age of ESRD onset in blacks (43.2 versus 55.4 years) [10]. Abbott and Agodoa found that patients in the USRDS database with ADPKD were more likely to be Caucasian, renal transplant recipients, and younger [11].

Freedman et al. found no difference in age of ESRD onset and concluded that race should not be a risk factor for ESRD secondary to ADPKD [12]. A large US study analyzed data from the USRDS (*n* = 23,722) between the years 2001 and 2010. Patients with ESRD from ADPKD were significantly more likely to be white and non-Hispanic [13].

Despite the assumption that the incidence of ADPKD is equal between races, our review of the literature suggests most US patients reported to have the disease are white. In this study, we compare data from the USRDS and US census data to determine whether there is a difference in the incidence of ESRD from ADPKD between non-Hispanic blacks and non-Hispanic whites in the US. We then evaluated for any differences in the age of onset of ESRD in these populations.

## Methods

In this retrospective study, USRDS data was used to evaluate and compare the incidence of ESRD secondary to ADPKD, diabetes, or hypertension from 2004 through 2013. Because the identity of individual subjects is not disclosed and not obtainable from the USRDS, individual consent was not obtained. The online USRDS Renal Data Extraction and Referencing (RenDER) System from the USRDS Coordinating Center was used for the collection of population level data used for our analyses [14].

Cases of ESRD secondary to ADPKD were those within the USRDS with the primary cause of ESRD listed as “Polycystic kidneys, adult type (dominant)” (ICD-9 code 75313) on the End Stage Renal Disease Medical Evidence Report (form CMS-2728) and were identified by the USRDS Coordinating Center. Cases of ESRD from diabetes or hypertension were those within the USRDS with the primary cause of ESRD falling under the broader categories of “Diabetes” (ICD-9 codes 250.40 and 250.41) or “Hypertension/Large Vessel Disease” (ICD-9 codes 403.91, 404.01, 593.81) on form CMS-2728 and were identified using the RenDER system.

The available variables included race, defined as either non-Hispanic white or non-Hispanic black, and age at ESRD onset. Ages were grouped together until a count of > 10 patients was reached to avoid a protected health information breach, which yielded a youngest age group of < 40 years old and an oldest age group of ≥75 years old. The ten-year period used for data collection and analysis was defined as 2004 through 2013. Both USRDS and US Census data were used as population denominators for each year, with race defined as non-Hispanic white or non-Hispanic black.

The difference in the incidence of ESRD in black and white patients at each age categorical bin (e.g., < 40, 40–44, 45–49, …, 75+) was compared by two-sample proportion test. The Cochran-Mantel-Haenszel test was used to investigate the association of race and incidence of ESRD secondary to ADPKD, diabetes, or hypertension after taking into account the stratification factor of year (ranging from 2004 to 2013). Odds ratio and 95% confidence intervals (CI) were calculated to measure the magnitude of the association between race and incidence of ESRD attributed to each of the three primary disease diagnoses.

The age of ESRD onset between non-Hispanic black and non-Hispanic white patients at each calendar year (2004–2013) was compared using two-sample t-test with unequal variance. All the statistical analyses were two-sided and were performed using the statistical software SAS v9.4 (Cary, NC). A *p*-value of less than 0.05 was considered statistically significant.

## Results

**Fig. 1:**
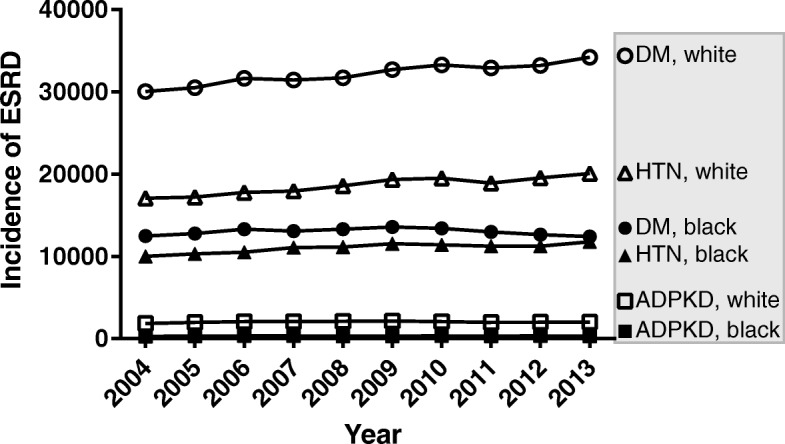
Incidence of ESRD with a primary diagnosis of diabetes mellitus (DM; circles), hypertension (HTN; triangles), or ADPKD (squares) from 2004 through 2013. Non-Hispanic whites are represented by open markers and non-Hispanic blacks are represented by solid markers. Source: USRDS database

We looked at proportions of non-Hispanic blacks and non-Hispanic whites with incident ESRD secondary to ADPKD, HTN or diabetes in the USRDS database. For the combined years examined (2004 through 2013), there were a total of 771,759 non-Hispanic whites and non-Hispanic blacks with incident ESRD with primary disease diagnoses of ADPKD (*n* = 23,647), diabetes (*n* = 451,760), or hypertension (*n* = 296,352) (Fig. 1). From this, we derived an average ten-year incidence (n per 100,000) of ESRD from each of the three primary disease diagnoses (Table 1). The numerator is the USRDS data, the denominator is the US Census data for each racial group. Non-Hispanic blacks compared to non-Hispanic whites were found to have significantly higher incidence of ESRD secondary to diabetes (34.508 (34.321–34.697) per 100,000 vs. 14.394 (14.344–14.444) per 100,000, *p* <  0.0001), hypertension (29.287 (29.115–29.461) per 100,000 vs. 8.322 (8.282–8.360) per 100,000, p <  0.0001), but lower incidence of ESRD secondary to ADPKD (0.864 (0.852–0.875) per 100,000, vs. 0.912 (0.909–0.916) per 100,000, p <  0.0001).

**Table 1:** Average (95% CI) 10-year incidence (N per 100,000) of ESRD for each primary diagnosis in the general non-Hispanic Black and non-Hispanic White populations

Race	ADPKD, N per 100,000	DM, N per 100,000	HTN, N per 100,000
Black	0.864 (0.852–0.875)	34.508 (34.321–34.697)	29.287 (29.115–29.461)
White	0.912 (0.909–0.916)	14.394 (14.344, 14.444)	8.322 (8.284–8.360)
Black vs. White	− 0.048 (− 0.060 – − 0.037)	20.114 (19.920–20.308)	20.966 (20.789–21.143)
*p-value*	*< 0.0001*	*< 0.0001*	*< 0.0001*

Denominator: US Census

**Fig. 2:**
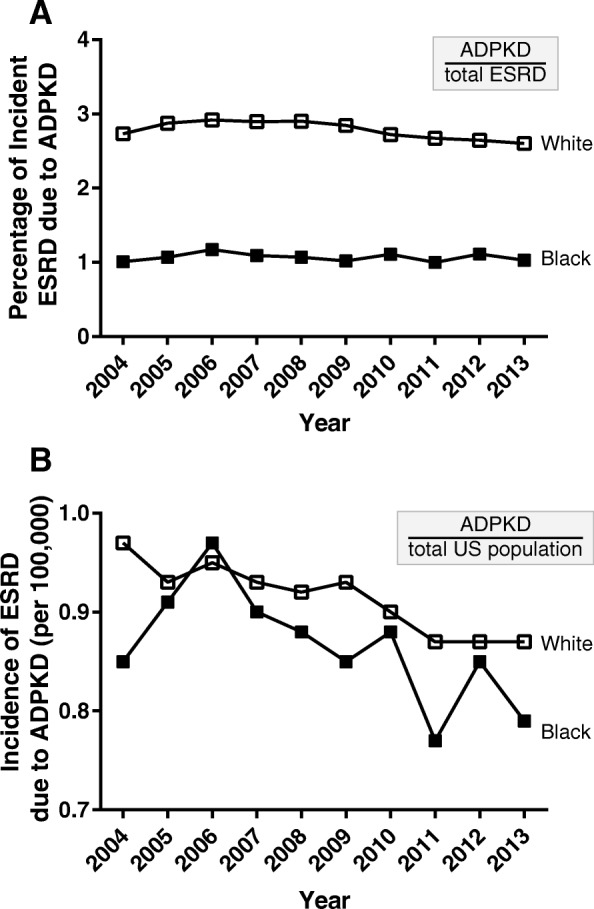
**a** Percentage of individuals with incident ESRD with a primary diagnosis of ADPKD in the USRDS database. **b** Incidence of ESRD due to ADPKD in the USRDS database per 100,000 people in the general United States population (Census data). The analysis included those who identified as non-Hispanic white (open squares) and non-Hispanic black (solid squares) from 2004 through 2013

**Fig. 3:**
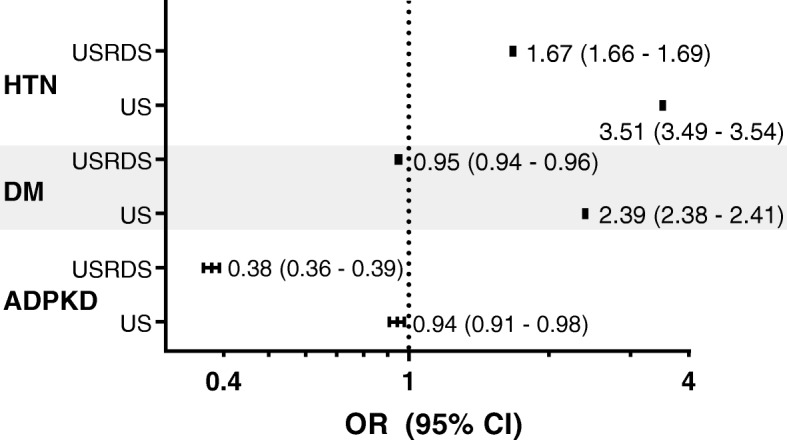
Odds Ratio (OR) and 95% Confidence Interval (CI) for non-Hispanic blacks compared to non-Hispanic whites for incident ESRD due to hypertension (HTN), diabetes mellitus (DM), or ADPKD. Odds ratios were calculated using the number of ESRD patients in the USRDS database as the numerator and either the total USRDS database (USRDS) or the total US Census population (US) as the denominator. The point estimate is given with the CI shown in parentheses. The *p* value was < 0.0001 for all groups except for the analysis of ADPKD in the general US population, which had a *p* value of 0.004

When considering only the USRDS population, similar trends were also identified, e.g., the average 10-year incidence of ESRD secondary to ADPKD was significantly lower in non-Hispanic black vs. non-Hispanic white (Fig. 2). After adjusting for the stratification factor of year, we found that blacks were less likely than whites to develop ESRD secondary to ADPKD (OR (95% CI) = 0.38 (0.36–0.39), *p* < 0.0001) and diabetes (OR (95% CI) = 0.95 (0.94–0.96), p < 0.0001). However, non-Hispanic blacks were more likely to have ESRD secondary to hypertension (OR (95% CI) = 1.67 (1.66–1.69), *p* < 0.0001) (Fig. 3, USRDS rows).

After normalizing the USRDS data for the differences in the proportion of non-Hispanic whites and non-Hispanic blacks in the US population as determined by US Census data, the risk of ESRD from ADPKD in non-Hispanic blacks was still lower (OR (95% CI) = 0.94 (0.91–0.96), *p* = 0.0004) (Fig. 3, US rows). The risk of ESRD secondary to hypertension in non-Hispanic blacks was higher (OR (95% CI) = 3.52 (3.49–3.54), p < 0.0001) as was the risk of risk of having ESRD secondary to diabetes (OR (95% CI) = 2.39 (2.38–2.41), p < 0.0001) (Fig. 3, US rows).

**Fig. 4:**
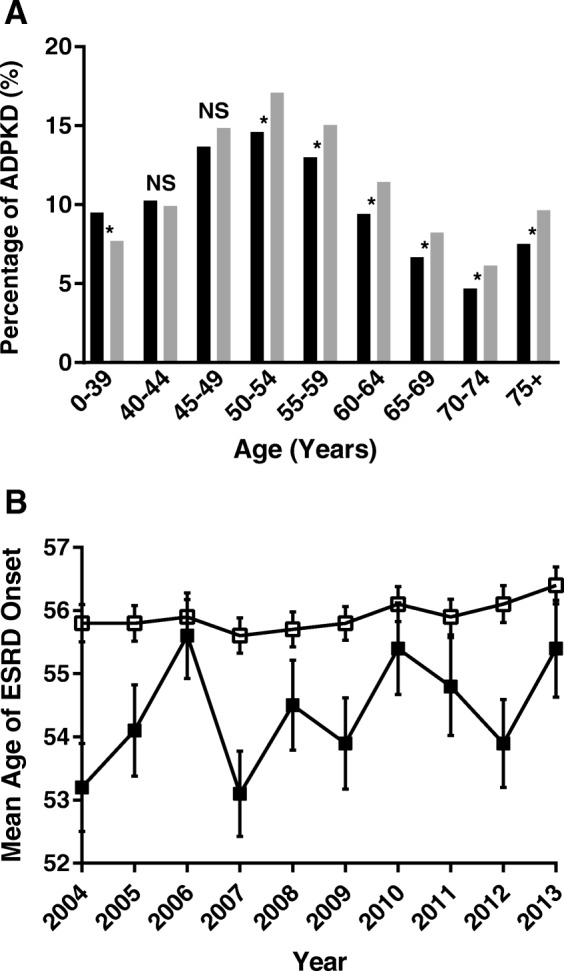
**a** Percent of ADPKD in incident ESRD, 2004–2012. Non-Hispanic blacks, black bars; non-Hispanic whites, gray bars. The p value was ≤0.002 for all groups with an asterisk (*) and was non-significant (NS) for the 40–44 and 45–49-year age groups, which had a p value of 0.53 and 0.065, respectively. **b** The mean age of ESRD onset for each year with standard error shown. Non-Hispanic whites, open squares; Non-Hispanic blacks, solid squares

These data were further analyzed to determine the age of onset of ESRD from ADPKD. For the lowest age group (< 40 years), there were significantly more blacks with ESRD secondary to ADPKD than whites (9.49% vs. 7.68%, difference (95% CI) = 1.81% (0.87–2.84%), *p* < 0.001) for the combined years examined (Fig. 4a).

Finally, we considered the mean age of initiation of dialysis for non-Hispanic blacks and non-Hispanic whites with ADPKD for each year between 2004 and 2013. The difference in the age of dialysis initiation due to ADPKD was significantly different (*p* < 0.05) in all age groups except for age 40–44 and age 45–49. The age of onset was lower for non-Hispanic black patients for each of the ten years Fig. 4b). Overall, blacks with ADPKD had a significantly lower age of initiation of dialysis (54.4, SD = 13) than whites with ADPKD (55.9, SD = 12.8; *p* < 0.0001).

## Discussion

As others have documented previously [11, 13], we found that non-Hispanic blacks are less likely to develop ESRD attributed to ADPKD than non-Hispanic whites. Further analysis of these data lead to a novel finding. In the youngest age group (< 40 years old) significantly more non-Hispanic blacks had incident ESRD attributed to ADPKD than young, non-Hispanic whites. Furthermore, non-Hispanic blacks with ADPKD progress to ESRD at an earlier age than non-Hispanic whites.

The prevalence of chronic kidney disease is higher in non-Hispanic blacks than in non-Hispanic whites [15]. In addition, blacks are three to four times more likely to progress to ESRD than whites [16].

Numerous studies have explored factors that contribute to health differences between races. Differential access to health care or health insurance [17], health literacy [18], physician beliefs and biases [19], and residential segregation commonly found in urban areas [20] are factors shown to have an impact on renal outcomes in blacks. While socioeconomic status has been shown to negatively impact blacks more than whites who progress to ESRD [21], these effects may be less relevant in ADPKD.

Socioeconomic status was not associated with higher risk of ESRD in black men or women with ADPKD [22] although it was for diabetes mellitus and for lupus nephritis. During the time of the study, 1996–2004, no effective therapy was available for slowing down progression to ESRD in ADPKD. The lack of effective therapy may have masked any underlying disparities due to socioeconomic status.

It is possible that ADPKD is underreported and/or underdiagnosed in blacks. A definitive diagnosis of ADPKD requires renal imaging. Since hypertension is more common in the black population, it is possible that young black patients with ADPKD and hypertension may never get a screening renal ultrasound. Blacks with ADPKD are less likely to have additional family members with ADPKD [12], which may lead to further under-diagnosis. Other co-morbidities commonly associated with ESRD such as hypertension, diabetes mellitus, sickle cell trait, or sickle cell disease could further mask the diagnosis of ADPKD and lead to differential attribution of the cause of ESRD.

Genetic factors may be contributing to the earlier age of ESRD in blacks with ADPKD compared to whites. Progression of ADPKD is associated with increased renal hypoxia and decreased renal blood flow [4]. Blacks are more likely to have sickle cell trait and it is known that blacks with sickle cell trait and ADPKD progress to ESRD about 10 years earlier than blacks with ADPKD without sickle cell trait [10]. Thus, factors which increase renal hypoxia may be additive in the progression of ESRD.

Apolipoprotein L1 (*APOL1)* risk alleles in blacks confer higher risk of ESRD from several causes [23]. Blacks have a higher prevalence of high-risk *APOL1* variants, which are associated with higher rates of ESRD, even among those with well-controlled blood pressures [24]. There are no studies evaluating a potential interaction of *APOL1*-related disease with ADPKD, therefore it is unknown whether the higher prevalence of *APOL1* risk alleles in blacks impacts the earlier age of onset of ESRD due to APDKD.

The strength of this study is in the fact that the USRDS captures virtually all ESRD patients in the US, which makes it possible to find an association potentially missed in smaller registries. However, our study has several limitations, first it is a retrospective review of the USRDS database. Patients who progress to ESRD but go to pre-emptive transplantation or chose to forego treatment are therefore not included. Since the rates of transplantation are higher in whites compared to blacks, and since younger patients are more likely to get pre-emptive transplants, the age of onset of ESRD may be more similar than we have reported. Because this was registry data it is possible that not all disease cases were captured since our use of the RenDER System did not allow for the gathering of individual patient data or family histories. Validating all cases of ESRD secondary to ADPKD requires diagnostic imaging, genotypic information, and family histories. Additionally, data may have been confounded due to underreporting or misclassification bias.

## Conclusions

Based on our analysis, non-Hispanic blacks compared to non-Hispanic whites with ADPKD may progress more rapidly to ESRD. Currently, there are two commonly used calculators, which are designed to predict progression to ESRD in ADPKD. The PROPKD score [6] was derived primarily from a Caucasian population in western France and uses genotype, sex, and the presence of a urologic event and/or hypertension before the age of 35 to assign a score which predicts age at onset of ESRD. The Mayo ADPKD classification calculator [8] uses htTKV age, race, and sex to predict time to decline in eGFR. Race is included only as needed to calculate eGFR and not as a factor for ADPKD disease progression. The Mayo cohort included relatively few blacks, potentially masking an effect of race due to small sample size.

Since it is likely that the PROPKD and/or the Mayo image classification score will be increasingly used to determine risk of progression in ADPKD, it may be prudent to consider black race as an additional possible risk factor until ADPKD studies with larger numbers of blacks are available.

## Abbreviations

(PROPKD) score: The predicting renal outcomes in ADPKD
ADPKD: Autosomal dominant polycystic kidney disease
CRISP: the Consortium for Radiologic Imaging Studies of Polycystic Kidney Disease
eGFR: estimated glomerular filtration rate
ESRD: End Stage Renal Disease
HALT PKD: The Halt Progression of Polycystic Kidney Disease
HTN: Hypertension
htTKV: Height-adjusted total kidney volume
USRDS: United States Renal Data System
